# Editorial: Immunometabolic mechanisms underlying the severity of COVID-19

**DOI:** 10.3389/fimmu.2022.977907

**Published:** 2022-07-20

**Authors:** Julia Kzhyshkowska, Vishwanath Venketaraman, Galileo Escobedo

**Affiliations:** ^1^ Institute of Transfusion Medicine and Immunology, Institute for Innate Immunoscience (MI3), Medical Faculty Mannheim, Heidelberg University, Mannheim, Germany; ^2^ German Red Cross Blood Service Baden-Württemberg – Hessen, Mannheim, Germany; ^3^ Laboratory for Translational Cell and Molecular Biomedicine, Tomsk State University, Tomsk, Russia; ^4^ Western University of Health Sciences College of Osteopathic Medicine of the Pacific-Pomona, Pomona, CA, United States; ^5^ Laboratory of Immunometabolism, Research Division, General Hospital of Mexico “Dr. Eduardo Liceaga”, Mexico City, Mexico

**Keywords:** SARS-CoV-2, COVID-19, type 2 diabetes, immune cell, cytokine storm, immunometabolism, anti-SARS-CoV-2 vaccination, COVID-19 sequelae

The severe acute respiratory syndrome coronavirus-2 (SARS-CoV-2) is the causative agent of the global outbreak of coronavirus disease 2019 (COVID-19) (1). A substantial number of COVID-19 patients developed the most severe form of the disease, including pneumonia, acute respiratory distress syndrome (ARDS), multiorgan failure, and death (2). COVID-19 caused more than six million deaths worldwide, with a higher prevalence in patients with preexisting comorbidities such as obesity, hypertension, type 2 diabetes (T2D), and coronary heart disease (CHD) (3). Patients with these preexisting conditions displayed a cytokine storm characterized by increased levels of proinflammatory cytokines such as interleukin (IL-) 1 beta, IL-6, and tumor necrosis factor-alpha (TNF-alpha), accompanied by an impaired T and B cell-mediated immunity (4, 5). However, the mechanisms through which metabolic and cardiovascular comorbidities hinder the antiviral immune response and enhance the cytokine storm, increasing the progression, severity, and mortality of COVID-19, are not yet fully understood. For this reason, we edited a Research Topic within Frontiers in Immunology to examine how immunometabolic agents such as excess glucose, adipose-derived hormones, and lipids act in synergy with immune cells to worsen the course of COVID-19.

We received numerous high-quality submissions from eminent research teams worldwide focused on different aspects of the crosstalk between immunity and metabolism in the COVID-19 setting.

Guo et al. contributed to the Research Topic by presenting original data comparing the production of serum cytokines belonging to the cytokine storm-related inflammatory response or the T cell-mediated adaptive immunity. They found that severity in COVID-19 patients increased as IL-6 soared and IL-2 decreased, indicating a biphasic trend where cytokines involved in the T cell-mediated adaptive immunity lose relevance while the inflammatory response gains it in severe COVID-19. The T cell-mediated adaptive immunity involves multiple cell types, among which CD3+CD4+ and CD3+CD8+ T cells and natural killer T (NKT) cells play crucial antiviral functions. Mo et al. expanded on this body of evidence by demonstrating that T lymphocytopenia often observed in patients with severe COVID-19 is hallmarked by a considerable reduction in the number of mitochondria in CD3+CD4+ and CD3+CD8+ T cells and poor lymphocyte response. In turn, using single-cell RNA sequencing, Yang et al. demonstrated that NKT cells expressing Tim-3 display increased apoptosis and exhaustion marker expression such as caspase-3 and PD-1, leading to NKT cell depletion and COVID-19 worsening.

At this point, contributions made to the Research Topic consistently show that impairment in immune cells with primary antiviral functions such as helper and cytotoxic T lymphocytes and NKT cells plays a crucial role in COVID-19 severity. The underlying mechanisms through which the immunometabolic agents exert their effects on these immune cells are also covered in this Research Topic. Tyurin et al. conducted a study in a cohort of COVID-19 patients and found that cardiovascular risk factors such as hypertension and coronary artery disease hallmark the progress of COVID-19. This research team described that those cardiovascular conditions boost COVID-19 severity by reducing the number of functional helper and cytotoxic T lymphocytes and B cells and increasing the amount of natural killer (NK) cells. Disbalance in the adaptive and innate cellular content correlated with circulating C-reactive protein (CRP) levels and suppressed iron metabolism. Singh et al. wrote a review manuscript examining how type 2 diabetes (T2D) predisposes to developing severe COVID-19 by inducing the release of IL-6, TNF-alpha, and CRP while decreasing regulatory cytokines such as IL-10. This immune scenario favored by excess glucose and free radical production is crucial to promoting the cytokine storm associated with the most severe form of the SARS-CoV-2 infection. Therefore, the use of agents such as glutathione and vitamin D may offer novel therapeutic avenues to treat COVID-19 patients due to their ability to decrease the production of proinflammatory cytokines and free radicals.

Furthermore, using single-cell RNA sequencing, Shao et al. demonstrated that T cells expressing the C-C motif chemokine ligand 2 (CCL2), secreted phosphoprotein 1 (SPP1)-producing macrophages, and dendritic cells (DC) are enriched in bronchoalveolar lavage fluid of patients with severe COVID-19. The authors found that increased glycolysis, fatty acid metabolism, bile acid synthesis, and purine and pyrimidine metabolism may favor CCL2 and SPP1 expression, predisposing the patient to develop the cytokine storm. Rendeiro et al. extended this body of evidence by conducting a study to characterize the serum metabolome of COVID-19 patients through high-throughput targeted nuclear magnetic resonance (NMR) spectroscopy and high-dimensional flow cytometry. They informed that immunometabolic agents involved in dyslipidemia, such as low-density lipoproteins (LDL) and very low-density lipoproteins (VLDL), act in synergy with NK cells to induce Tim-3 expression and increase the severity of COVID-19. Of note, the authors also showed additional benefits of studying the crosstalk between metabolism and immunity by using VLDL and Tim-3 as potential predictors of response to tocilizumab in the treatment of COVID-19. Similarly, resistin is a hormone secreted by adipose tissue and mononuclear leukocytes with crucial roles in obesity, insulin resistance, and inflammation. Ebihara et al. made a valuable contribution to the Research Topic by showing that serum resistin increases as COVID-19 severity raises and could be an accurate mortality predictor in COVID-19 patients. The authors proposed that resistin can display these actions because it is associated with a cytokine storm where IL-6, IL-8, IL-10, and CCL2, and the endothelial damage markers intercellular adhesion molecule-1 (ICAM-1) and vascular cell adhesion molecule-1 (VCAM-1) considerably elevate.

As outlined here, immunometabolic agents such as excess glucose, VLDL, and resistin, among others, can activate immune cells and promote cytokine release, contributing to an impaired adaptive immune response that allows the cytokine storm, which in turn worsens the severity of COVID-19. However, additional papers published in the Research Topic taught us that the synergy between immunometabolic agents and immune cells goes beyond the effects of carbohydrates and lipids on T and B lymphocytes and NK cells. In this sense, Beltrame et al. conducted a study on 138 patients hospitalized due to COVID-19. They observed that decreased testosterone blood levels and increased estradiol values were associated with ARDS onset and increased mortality, mainly in males. Thus, unbalanced testosterone and estradiol levels may increase the risk of developing severe COVID-19, probably due to differential hormonal effects on immune cells. Moreover, Chakraborty et al. used the Agilent-085982 Arraystar human lncRNA V5 microarray and found multiple genes expressed differentially in mild and severe COVID-19 patients, including endothelin 1 (EDN1) and ribosomal protein L19 (RPL19). EDN1 and RPL19 belong to the TLR4 and TLR3-dependent pathways that confer protection against viral infections through signaling in antigen-presenting cells such as DCs. Chang et al. expanded on this body of evidence by writing a review article where they summarize the primary mechanisms through which DCs contribute to COVID-19 progression by showing a reduced number, impaired antigen-presentation ability, and low type-I interferon release. The authors also examined interesting evidence indicating that designing effective anti-SARS-CoV-2 drugs and vaccines should consider enhancing DC activity against the virus, strengthening the link between innate and adaptive immune responses without soaring the cytokine storm.

The most severe form of COVID-19 includes thrombosis and multiorgan failure that increase the risk of intensive care unit (ICU) admission and in-hospital death. Zou et al. conducted a bioinformatics analysis based on a time-order gene co-expression network (TO-GCN). The authors reported the expression of numerous immune genes (i.e., SRC, RHOA, CD40LG, CSF1, TNFRSF1A, FCER1G, ICAM1, LAT, LCN2, PLAU, CXCL10, ICAM1, CD40, IRF7, and B2M, among others) that mediate leukocyte overactivation and promote the cytokine storm in COVID patients who developed multiorgan failure. Hartmann et al. extended this body of information by conducting an extensive postmortem immunohistochemical study in myocardium biopsies of patients who died from COVID-19 to understand how the disease drives heart failure. The study revealed that myocardial injury results from local overexpression of IL-1 beta, IL-6, and TNF-alpha, which is associated with interstitial edema, endothelial cell apoptosis, and increased TGF-beta 1 expression, leading altogether to chronic myocardial fibrosis. This work also supports that clinical surveillance is critical in patients recovering from severe COVID-19 to detect myocardial dysfunction and arrhythmias as potential cardiac sequelae of the SARS-CoV-2 infection. Furthermore, Ebihara et al. performed an extensive proteomic analysis of more than a thousand plasma proteins, finding that patients with severe COVID-19 showed an elevation in IL-6 and growth differentiation factor (GDF)-15 like that found in patients with sepsis. Sepsis is a frequent complication in critically ill patients with COVID-19. Therefore, besides helping identify patients at higher risk of sepsis, measurement of GDF-15 may also allow for predicting a late recovery in COVID-19 cases admitted to ICU. Ahmed et al. published a review article that expands on the possible mechanisms by which GDF-15 contributes to COVID-19 aggravation, including mitochondrial stress, free radical production, and inflammation. The information provided by the authors supports using GDF-15 as a potential biomarker of COVID-19 severity and for designing novel therapeutic interventions against the SARS-CoV-2 infection.

Thrombosis is also an often complication of patients hospitalized with severe COVID-19. In this sense, Komi et al. contributed to the Research Topic by conducting a study on a large cohort of COVID-19 patients admitted to ICU. The authors studied the coagulation cascade’s dynamic changes, observing that D-dimer, fibrinogen, and prothrombin time are essential in developing a pro-thrombotic state that aggravates the course of COVID-19 until the critical stage. Pastorek et al. extended these mechanisms by writing a mini-review where they summarize the most relevant signals inducing neutrophil extracellular trap (NET) formation, including Toll-like receptor (TLR) 2, 4, 7, 8, and 9 and other pattern recognition receptors (PRR). The authors examined the possible role of the complement activation in allowing thrombin release, which promotes tissue factor (TF) activity that can stimulate NET formation and thrombosis in COVID-19. Among other aspects, this article may contribute to understanding the apparent relationship between NETs and the occurrence of ischemic stroke in patients with severe SARS-CoV-2 infection.

In addition to covering the mechanisms involved in severe COVID-19 and the complications affecting several organs, this Research Topic also received valuable contributions to drug development research, especially by using old drugs with novel therapeutic applications against the SARS-CoV-2. Cory et al. reported that exposure of primary human monocytes to the recombinant SARS-CoV-2 spike protein subunit 1 provoked a marked metabolic reprogramming consisting of increased glycolytic metabolism and release of IL-1 beta, IL-6, and TNF-alpha. Notably, pretreatment of monocytes with metformin inhibited the glycolytic overactivation and inflammatory cytokine release in response to either recombinant spike S1 protein or SARS-CoV-2 strain WA1/2020 *in vitro*. This report provides solid evidence that allows thinking on the feasibility of testing metformin in prospective clinical trials to prevent the cytokine storm associated with hyperactivation of immune cells observed in severe COVID-19. Moreover, this study highlights the essential role of innate immunity in the detrimental amplification of inflammation during acute COVID-19 and the possibility of reverting exacerbation of innate immune reaction by targeting their metabolism. Likewise, Teixeira et al. performed an experimental study using a strategy that combined *in vivo* infection assays and *in vitro* cultures. They found that simvastatin decreased viral replication and lung damage in the K18-hACE2-transgenic mouse model infected with the SARS-CoV-2 gamma strain. *In vitro*, simvastatin reduced NET formation and release of TNF-alpha and CCL5 in neutrophils, and the production of TNF-alpha, IL-6, and IL-8 in monocytes *via* lipid raft disruption, which may hinder viral adhesion to target cells and reduce viral replication. Dai et al. extraordinarily complemented this information, writing a review manuscript to examine how statins inhibit adhesion and binding of the SARS-CoV-2 to target cells. They propose blockage of cholesterol synthesis by statins also promotes lipid raft disruption, decreasing the ability of the SARS-CoV-2 to adhere to the host cell’s plasma membrane, leading to lessening viral replication and load. The authors also discuss evidence showing that statins lower the levels of IL-6 and TNF-alpha, two inflammatory cytokines essential to developing the cytokine storm, making these ideal drug candidates for treating patients seriously ill with COVID-19. Altogether, this evidence confirms the hypothesis postulated by Elkoshi, who applied a binary model of chronic diseases to COVID-19 and proposed that simvastatin and lovastatin could be more effective in treating patients with severe SARS-CoV-2 infection. According to Elkoshi’s hypothesis, the main reason behind suggesting statins as promising drugs for treating COVID-19 is that they may induce a robust regulatory T cell activity and vigorous CD3+CD8+ T cell function, strengthening the adaptive arm of immunity and reducing the innate inflammatory response.

Finally, this Research Topic also centered the study of immunometabolism on several aspects we should pay attention to during the post-epidemic era, such as COVID-19 sequelae. Yang et al. collected information from more than a thousand articles and systematically analyzed the most frequent COVID-19 sequelae. They reported that the most common sequelae in patients who recovered from COVID-19 are lung fibrosis resulting in cough, shortness of breath, and dyspnea. The authors also found myocarditis resulting in ischemia, elevated blood pressure, and increased afterload as frequent COVID-19 sequelae. They also informed that brain bleeding, cranial nerve injury, and temporary loss of consciousness are often found after severe COVID-19 illness. In parallel, Jing et al. characterized the urinary metabolome of 248 patients with several COVID-19 severities, revealing that oxoglutaric acid, indoxyl, and phenylacetamide were prominently associated with low helper and cytotoxic T cell count and decreased serum levels of IFN-gamma and IL-2. Interestingly, the authors also reported that those urinary metabolites allowed to predict the occurrence of memory deterioration and other psychiatric sequelae after COVID-19 recovery.

Another aspect we should be aware of during the post-pandemic era is paying attention to any adverse effects of anti-SARS-CoV-2 vaccines, above all those using adenoviral non-replicating vectors (AVV). Azzarone et al. wrote a perspective article delineating a mechanism leading to vaccine-induced immune thrombotic thrombocytopenia (VITT), an uncommon, life-threatening syndrome occurring after the first injection of AVV anti-SARS-CoV-2 vaccines. The authors propose that VITT results from the interaction of AVV with platelets and mast cells that consequently release platelet factor 4 (PF4) and heparin, favoring PF4/heparin/IgG complexes that may enhance platelet activation and neosynthesized spike S1 protein-induced thrombotic events.

As we have outlined here, the Research Topic included a variety of seminal articles expanding on the mechanisms through which metabolic diseases and immunometabolic agents act in synergy with adaptive and innate immune responses to increase the severity of COVID-19. The findings published in our special issue also show the perspectives for optimizing the metabolism of immune cells to minimize the damage that SARS-CoV2 makes in the human body. We thank the authors for sharing their extraordinary research works that will open new avenues to understand the immunometabolic bases driving disease worsening, contribute to drug development research, and prevent complications and sequelae of COVID-19.

## Author Contributions

All authors listed have made a substantial, direct, and intellectual contribution to the work and approved it for publication.

## Conflict of Interest

The authors declare that the research was conducted in the absence of any commercial or financial relationships that could be construed as a potential conflict of interest.

## Publisher’s Note

All claims expressed in this article are solely those of the authors and do not necessarily represent those of their affiliated organizations, or those of the publisher, the editors and the reviewers. Any product that may be evaluated in this article, or claim that may be made by its manufacturer, is not guaranteed or endorsed by the publisher.
